# Effect of Wet-Dry Cycles on the Bond Behavior of Fiber-Reinforced Inorganic-Matrix Systems Bonded to Masonry Substrates

**DOI:** 10.3390/ma14206171

**Published:** 2021-10-18

**Authors:** Karrar Al-Lami, Angelo Savio Calabrese, Pierluigi Colombi, Tommaso D’Antino

**Affiliations:** 1 ABC Department of Architecture, Built Environment and Construction Engineering, Politecnico di Milano, 20133 Milan, Italy; karrar.allami@polimi.it (K.A.-L.); angelosavio.calabrese@polimi.it (A.S.C.); pierluigi.colombi@polimi.it (P.C.); 2 Civil Engineering Department, University of Wasit, Al-Rabee St., Wasit 00964, Iraq

**Keywords:** wet-dry, FRCM, CRM, SRG, masonry, durability, direct shear test, bond

## Abstract

In recent years, inorganic-matrix reinforcement systems, such as fiber-reinforced cementitious matrix (FRCM), composite-reinforced mortars (CRM), and steel-reinforced grout (SRG), have been increasingly used to retrofit and strengthen existing masonry and concrete structures. Despite their good short-term properties, limited information is available on their long-term behavior. In this paper, the long-term bond behavior of some FRCM, CRM, and SRG systems bonded to masonry substrates is investigated. Namely, the results of single-lap direct shear tests of FRCM-, CRM-, and SRG-masonry joints subjected to wet-dry cycles are provided and discussed. First, FRCM composites comprising carbon, polyparaphenylene benzobisoxazole (PBO), and alkali-resistant (AR) glass textiles embedded within cement-based matrices, are considered. Then, CRM and SRG systems made of an AR glass composite grid embedded with natural hydraulic lime (NHL) and of unidirectional steel cords embedded within the same lime matrix, respectively, are studied. For each type of composite, six specimens are exposed to 50 wet–dry cycles prior to testing. The results are compared with those of nominally equal unconditioned specimens previously tested by the authors. This comparison shows a shifting of the failure mode for some composites from debonding at the matrix–fiber interface to debonding at the matrix-substrate interface. Furthermore, the average peak stress of all systems decreases except for the carbon FRCM and the CRM, for which it remains unaltered or increases.

## 1. Introduction

Inorganic-matrix composites represent a relatively new solution for strengthening and retrofitting existing reinforced concrete (RC) and masonry structures. They are based on the same principles of fiber-reinforced polymer (FRP) composites, where high-strength fiber sheets are coupled with polymeric matrices. However, in inorganic-matrix composites, the polymeric binder is replaced by an inorganic matrix (typically a cement-based, lime-based, or geopolymer mortar [1,2,3]), which provides good compatibility with the substrate, vapor permeability, and resistance to high temperature. Depending on the fiber and matrix type employed, inorganic-matrix composites can be referred to as fiber-reinforced cementitious matrix (FRCM) or textile-reinforced mortar (TRM), where open-mesh textiles and cement- or lime-based mortars are employed [4,5] (in this paper, the acronym FRCM is adopted), textile-reinforced concrete (TRC), where high strength finely grained concrete embeds open-mesh textiles [6,7], or steel-reinforced grout (SRG), which are comprised of unidirectional steel cords and inorganic matrices [8,9]. Recently, systems made of composite grids embedded within inorganic matrices, which are referred to as composite-reinforced mortar (CRM), have been increasingly adopted as externally bonded reinforcement of masonry members [10]. CRM systems are particularly attractive because of their simplicity of installation and low price [11].

Inorganic-matrix systems can be comprised of different types of fiber, e.g., glass, carbon, basalt, polyparaphenylene benzobisoxazole (PBO), and steel. Depending on several parameters, such as the textile layout, textile/grid equivalent thickness and spacing, and matrix type, a peculiar physical and mechanical behavior is obtained. In general, carbon and PBO FRCM and SRG systems have a high tensile capacity, while glass and basalt FRCM and glass CRM systems have a lower tensile capacity. Their different performances and behavior can be exploited to properly design the strengthening/retrofitting application depending on the specific case [12]. 

Inorganic-matrix composites and CRM systems showed promising results in increasing the bearing and displacement capacity of masonry members [13,14,15], preventing slab intrados crumbling hazards [16], and increasing the fatigue life of structural members subjected to cyclic loading [17]. However, the effectiveness of externally bonded (EB) inorganic-matrix reinforcement is strictly connected to the bond between the matrix and internal reinforcement and between the matrix and substrate. Accordingly, the investigation of inorganic-matrix reinforcement bond properties has gained increasing attention over the past decade [18,19]. Nonetheless, limited information is available on the durability of these reinforcing materials and on their bond properties [4]. 

Studies available in the literature focused on the effect of freeze–thaw cycles and saline and alkaline environments on the tensile capacity of FRCM coupons. Among them, Arboleda [20] investigated the effect of freeze–thaw cycles, saline solution (seawater), and alkaline solution on the tensile capacity of FRCM coupons including carbon and PBO textiles according to the recommendations of AC434 [21]. Results indicated a slight increase (approximately 10%) in the tensile capacity of PBO FRCM coupons after 20 freeze-thaw cycles and 1000 h of immersion in seawater, while carbon FRCM coupons showed no significant variation after freeze–thaw cycles and an increase of approximately 13% after 1000 h of immersion in alkaline solution. Similarly, Donnini et al. [22] investigated the effect of freeze–thaw cycles and saline and alkaline environments on the tensile capacity of FRCM coupons made of AR glass textile and cement-based mortar. While no significant variation was observed after 40 freeze–thaw cycles, a slight increase of tensile capacity was observed after 1000 h of conditioning in saline and alkaline solutions. Nobili [23] studied the effect of saline and alkaline solutions on the tensile capacity of an AR glass FRCM and observed reductions in the range of 10% to 15% after 1000 h of conditioning depending on the type of matrix. Similar tensile capacity decreases were observed by Colombo et al. [24] and De Munck et al. [25], which exposed AR glass TRCs to 25–500 and 100 freeze–thaw cycles, respectively. 

Studies dedicated to investigating the durability of the bond between inorganic-matrix reinforcement and specific substrates are quite limited. Donnini et al. [2] exposed AR glass FRCM-masonry joints to 10 wet–dry cycles in saline solution and observed a 20% reduction in their peak stress. In addition, the failure mode was shifted from the matrix–fiber interface to the matrix–substrate interface. Franzoni et al. [1] observed a 16.3% reduction of peak stress of SRG-masonry joints subjected to 6 wet–dry cycles in saline solution, while a 12% reduction was obtained when the same cycles were performed in deionized water. 

The results available in the literature does not allow for identifying a clear trend regarding the effect of various environmental exposures and accelerated aging. Furthermore, the limited information on the long-term bond behavior of FRCM, SRG, and CRM systems might limit their utilization or force to use quite severe environmental conversion factors [26]. In this paper, the long-term bond behavior of inorganic-matrix reinforcements is investigated by exposing FRCM-, SRG-, and CRM-masonry joints to 50 wet–dry cycles and then testing them using a single-lap direct shear test set-up. The FRCM composites comprised carbon, PBO, and AR glass textiles embedded within cement-based matrices, while the CRM and SRG comprised an AR glass composite grid and unidirectional steel cords, respectively, embedded within the same lime mortar. The exposure condition was designed to simulate a 25-year-long service life of externally bonded reinforcements that were fully soaked twice a year. This condition may be representative of the intrados of bridges subjected to cyclic floods [27]. The results obtained were compared with those of nominally equal unconditioned specimens previously tested by the authors [11,28].

## 2. Experimental Program

In this study, five inorganic-matrix reinforcement systems were studied, namely a carbon FRCM, a PBO FRCM, an AR glass FRCM, an SRG, and an AR glass composite grid CRM. Six specimens were prepared for each type of reinforcement and were all subjected to wet–dry cycles prior to testing. Nominally equal unconditioned specimens were presented and discussed in [11,28] and are considered here for comparison.

Specimens presented in this paper were named following the notation DS_X_Y_M_W/D_n, where DS is the test type (=direct shear), X and Y indicate the length and width of the composite strip in mm, respectively, M is the reinforcement type (C = carbon, P = PBO, G = AR glass, S = SRG, and CRM = composite-reinforced mortar), W/D (=wet/dry) indicates the conditioning, and n is the specimen number.

### 2.1. Materials and Methods

In this section, the main physical and mechanical properties of the matrix and reinforcement used are provided. Although these properties do not allow for directly obtaining indications on the matrix–fiber interaction, they are fundamental to understand the reinforcing system behavior and its failure mode. Table 1 reports the main geometrical and mechanical properties of the fiber reinforcements and matrices used in the five systems investigated. In Table 1, *b_f_*, *t_f_*, and *A_f_* are the width, thickness, and cross-sectional area of a single bundle (also referred to as yarn) along the warp direction, respectively. For steel cords and AR glass bundles, which are idealized with a circular cross-section, the cross-section diameter *d_f_* is provided. The tensile strength *f_f_* and elastic modulus *E_f_* of the bare fiber reinforcement (i.e., not embedded in the inorganic matrix) are also reported in Table 1. The mechanical properties of the inorganic binder, namely the compressive strength, flexural strength, and elastic modulus are also indicated in Table 1 as *f_c_*, *f_r_*, and *E_c_*, respectively. 

The carbon textile was a bi-directional balanced fiber open-mesh grid (i.e., same fiber amount in weft and warp direction), with bundles spaced at 10 mm on center in both weft and warp direction [29]. The PBO textile was a bidirectional unbalanced PBO open-mesh grid, with warp and weft yarns spaced at 10 mm and 17.5 mm on center, respectively [30]. Both carbon and PBO textiles were embedded within the same cement-based matrix [30]. The mechanical properties of carbon and PBO textiles were measured in [13,18], while those of the matrix in [13]. The AR glass FRCM composite was made by a coated AR glass open-mesh bidirectional textile embedded within a cement-based matrix [31] (Table 1). The textile bundles were spaced at 17 mm on center both in longitudinal and transversal direction. The fiber cross-sectional area in the textile warp direction was measured using the calcination method according to [32] and resulted slightly different from that reported in [28]. The tensile strength and elastic modulus of the AR glass textile were obtained by tensile testing of specimens comprising three longitudinal yarns according to [33] (Table 1). 

The SRG system was composed of unidirectional stainless-steel cords [34] embedded within a natural hydraulic lime 5 (NHL 5 [35]) mortar [36]. The cords were spaced at 5 mm on center and their mechanical properties were declared by the manufacturer (Table 1). Finally, the CRM comprised a bidirectional AR glass composite grid, made of pultruded yarns in the weft direction and twisted laminated yarns in the warp direction, weaved together using the leno weave technique [37]. The grid was spaced at 40 mm in both directions and was embedded within the same NHL 5 mortar used in the SRG [36,38]. The mechanical properties of the grid and mortar were evaluated in [11]. 

Clay brick [39] masonry blocks of dimensions 120 × 120 × 380 mm^3^ (width × thickness × height) were used as substrate for FRCM- and SRG-masonry joints, whereas historical brick masonry walls of dimension 325 × 160 × 330 mm^3^ (width × thickness × height) were employed to realize CRM-masonry joints. The clay brick blocks were constructed with six half bricks and five 10 mm thick joints made by a cement-based mortar [40]. The historical brick blocks were constructed with six historical clay bricks [14] and five joints made by a lime-based mortar [36]. The bonded length of the FRCM and SRG composites was 300 mm, as recommended by the Italian acceptance criteria for inorganic-matrix composites [33]. The bonded length of the CRM strips was 290 mm, which is the maximum bonded length possible for the masonry blocks employed (the strip end shall not coincide with the masonry block edge to avoid wedge failure) and the same considered in [11] to study the bond behavior of CRM-masonry joints. The width of the composite strip *b*_1_ was selected to be a multiple of the warp yarn spacing and was equal to 50 mm for FRCM and SRG composites, whereas it was 120 mm for the CRM system. The number of warp bundles *n* in longitudinal direction (i.e., aligned with the load direction) included in the reinforcement strips was equal to 5 for carbon FRCM, 5 for PBO FRCM, 3 for AR glass FRCM, 7 for SRG, and 3 for CRM. The reinforcement strip loaded end was placed 35 mm far from the edge of the masonry block to avoid possible wedge failure. 

**Table 1:** Inorganic-matrix reinforcements geometrical and mechanical properties.

Characteristic	Component		**Carbon FRCM** [29,30]	**PBO FRCM** [30]	**AR Glass FRCM** [31]	**SRG** [34,36]	**CRM** [36,38]
Reinforcement geometry	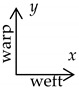		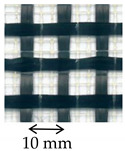	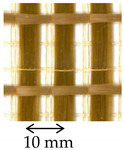	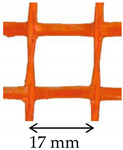	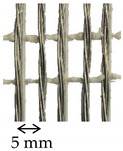	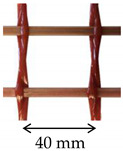
	warp yarn	*bf*	5 mm	5 mm	-	-	-
		*tf*	0.094 mm	0.092 mm	-	-	-
		*df*	-	-	1.16 mm	1.01 mm	-
		*Af*	0.47 mm^2^	0.46 mm^2^	1.05 mm^2^	0.80 mm^2^	5.71 mm^2^
Mechanical properties	Reinforcement	Type	Carbon fiber	PBO fiber	AR glass fiber	Steel cords	AR glass composite grid
		*ff*	1944 MPa	3014 MPa	874 MPa	>2000 MPa ^†^	817 MPa
		*Ef*	203 GPa	206 GPa	65 GPa	210 GPa ^†^	66 GPa
	Matrix	Binder	Cement-based	Cement-based	Cement-based	NHL 5	NHL 5
		*fc*	25 MPa	25 MPa	22 MPa ^†^	8.2 MPa	8.2 MPa
		*fr*	6.1 MPa	6.1 MPa	6.0 MPa ^†^	3.2 MPa	3.2 MPa
		*Ec*	>7.5 GPa ^†^	>7.5 GPa ^†^	7.6 GPa ^†^	-	-

^†^ Declared by the manufacturer.

Before applying the strips, the masonry block surface was wet using a painting brush to prevent the absorption of the matrix water. First, (internal) a 5-mm thick matrix layer [30,31,36] was applied to the masonry surface and the textile was gently pushed on it to promote proper impregnation. Then, a second (external) 5-mm thick matrix layer was applied over the textile. At the loaded end, the fiber reinforcement was left bare for approximately 300 mm. Furthermore, a 20 mm long portion of textile was left bare at the free end to monitor its displacement. This was not possible in the case of CRM due to the limited height of the masonry blocks. After applying the strips, the specimens were cured inside an environmental chamber at 25 °C and 90% RH for 28 days.

### 2.2. Wet–Dry Conditioning 

No standard or recommendation that provides the procedure for wet–dry conditioning is available in the literature. In many studies [2,41,42,43,44,45,46], lab tests were performed to determine the period needed to saturate and fully dry certain specimens exposed to specific environments and temperatures. For instance, Donnini et al. [2] and Franzoni et al. [45] considered 2 days and 8 h, respectively, for the wetting phase of FRCM-masonry joints, while the drying phase comprised 2 days in a ventilated oven at 60 °C. However, Yin et al. [46] tested FRCM coupons performing 12 h of wetting followed by 12 h of drying both at room temperature.

In this study, wet–dry cycles were performed using an automatic system made of two water containers connected by hoses. One container (inside container) was placed inside an environmental chamber while the other one (outside container) was left outside at lab temperature (approximately 25 °C). Two pumps controlled using timers were used to transfer the water from one container to the other at a specific time of the day. The specimens were placed in the inside container and exposed to 50 wet–dry cycles. The length of each cycle was 24 h, 6 of which for immersion in water and 18 for drying. The length of the wetting and drying phase was determined after performing trial tests on a masonry block to determine the period required to fully wet and dry the specimen. 

**Figure 1:**
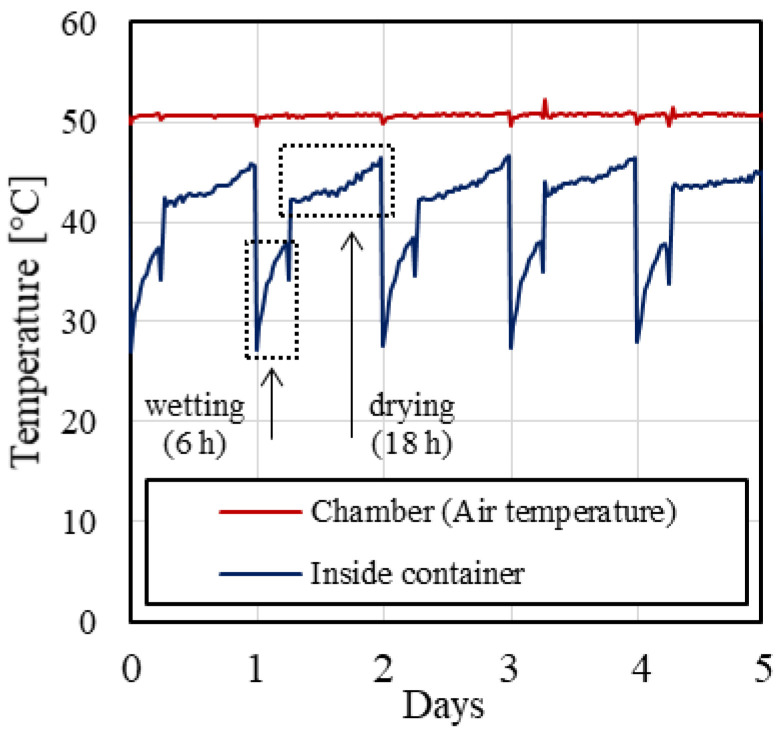
Temperature of environmental chamber and inside container during wet–dry cycles.

The temperature of the air inside the chamber was kept constant at 50 °C (Figure 1). The temperatures of the water wetting the specimens (i.e., in the inside container) and of the air inside the chamber were continuously monitored using thermocouples. The temperature in the inside container ranged between 27 °C at the beginning of the wetting phase and 38 °C at the beginning of the drying phase, when the inside container water was pumped out of the chamber. During the drying phase, the temperature of the air inside the chamber ranged between 42 °C and 47 °C from the beginning to the end of the drying phase, respectively (Figure 1). The drying temperature (50 °C) was selected to satisfy two criteria: (1) it is sufficiently high to allow for drying the specimens in 18 h and (2) it can simulate the temperature attained by the structure in real applications [45,47]. 

The number of cycles (i.e., 50 cycles) was selected to simulate a 25-year-long service life of externally bonded reinforcements that are fully soaked twice a year [27]. Furthermore, this specific number of wet–dry cycles is the same recommended by EN 12,467 [48] to investigate the durability of fiber-cement flat sheets, which confirms that it represents an adequate number of cycles for inorganic materials.

### 2.3. Direct Shear Single-Lap Test Set-Up

**Figure 2:**
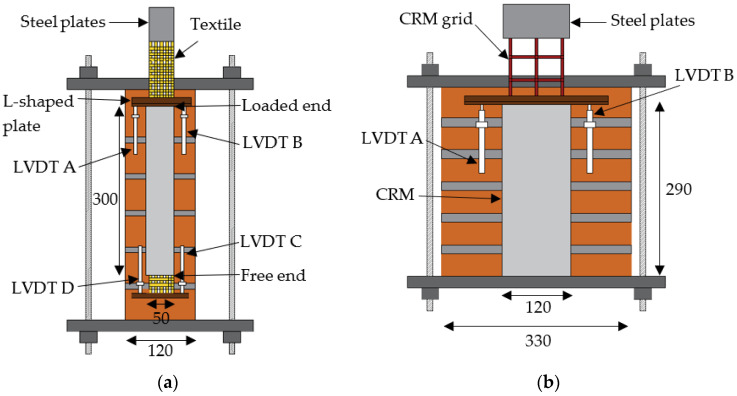
(**a**) Sketch of single-lap direct shear test set-ups used for (**a**) FRCM and SRG and (**b**) CRM (dimensions in mm).

The push-pull single-lap direct shear test set-up adopted in this study is shown in Figure 2. An MTS servo-hydraulic universal testing machine with a maximum capacity of 250 kN was employed. The specimens were restrained against movement using a steel frame comprised of two steel plates connected by four threaded bars. Two steel plates were epoxy bonded to the end of the bare fiber reinforcement at the free end to facilitate its gripping by the testing machine. The test was conducted in displacement (stroke) control mode with a rate of 0.2 mm/min [33]. The relative displacement between the bare textile just outside the matrix and the masonry substrate at the loaded end (Figure 2), referred to as the global slip *g*, was measured as the average of two linear variable displacement transducers (LVDT A and B) attached to the masonry on the sides of the reinforcement strip. The relative displacement between the bare textile just outside the matrix and the masonry substrate at the free end (Figure 2a), i.e., the free end slip *s_F_*, was measured as the average of LVDT C and D that were attached to the masonry on the sides of the reinforcement strip. Note that *s_F_* was measured only for one carbon FRCM-masonry joint and for all PBO and AR glass FRCM-masonry joints due to the complexity of the set-up. All the LVDTs reacted off of L-shaped aluminum plates glued to the bare textile at the free and loaded ends (Figure 2). 

## 3. Results and Discussion

The results obtained are reported in Table 2 for each specimen, where P* and σ* are the peak applied load and peak stress, respectively, whereas ${\bar{P}}^{*}$ and ${\bar{\sigma }}^{*}$ are the corresponding average peak applied load and peak stress for nominally equal specimens, respectively. σ is the ratio between the applied load *P* and the fiber reinforcement cross-sectional area *A* = *nA_f_*. Accordingly, σ* is the ratio between P* and *A.* In Table 2, specimens for which *s_F_* was measured are marked with the superscript § at the end of the name. 

**Figure 3:**
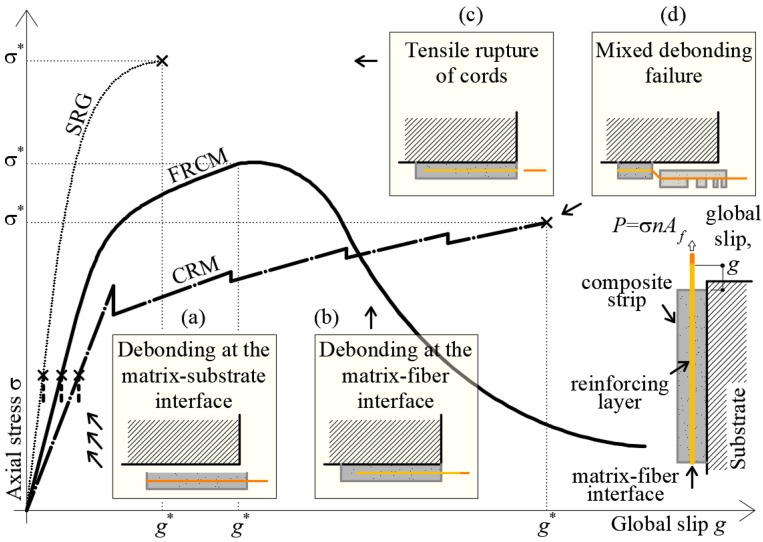
Representative axial stress–global slip responses of inorganic-matrix reinforcement-substrate joints subjected to direct shear single-lap test (typical failure modes are illustrated in boxes). σ* = peak axial stress; g* = global slip associated with σ*.

Figure 3 shows the representative axial stress σ—global slip *g* responses of the inorganic-matrix reinforcements investigated in this study.

When one or two layers of textile are employed, (dry) carbon, PBO, and AR glass FRCM-masonry (or concrete) joints generally fail due to debonding at the matrix–fiber interface (Figure 3) [5,18]. Their σ-*g* response is characterized by an initial linear branch, associated with the matrix–fiber interface elastic behavior, followed by a non-linear branch due to the occurrence of interface micro-cracking [19]. If the bonded length is sufficient to fully establish the bond stress transfer between matrix and fiber, with increasing the applied displacement, the axial stress increases until a unit crack forms at the loaded end and the debonding stress is attained. Beyond this point, the axial stress can further increase if friction/interlocking at the matrix–fiber interface and among fiber filaments is present [19], until the specimen peak stress σ*, which is associated with the global slip value g*, is reached. The extent of this branch of the response is related to the length of the bonded area. After this point, the propagation of debonding at the matrix–fiber interface determines a reduction of applied stress while the slip at the free end continues to increase. The applied stress eventually attains a constant value associated with pure friction/interlocking. Note that this behavior is the result of the stroke- or global slip-control mode adopted during the tests, which does not allow for capturing the snap-back phenomenon described by analytical and numerical models of the bond stress-transfer mechanism [19]. Nonzero values of the free end slip *s_F_* are recorded when the textile at the free end is engaged in the stress transfer mechanism, which occurs close to the attainment of σ*. 

SRG-masonry joints considered in this study generally failed due to debonding at the matrix-cord interface and rupture of steel cords [28]. The representative σ-*g* response of these joints (Figure 3) is characterized by a linear and a subsequent non-linear ascending branches. The former branch is associated with the elastic behavior of the matrix-cord interface, while the latter with the occurrence of micro-cracking and subsequent debonding at the same interface. The joint peak stress σ* is attained when the steel cords fail. In general, the applied load is not evenly distributed among the cords due to the random variation of matrix-cord interface properties, which leads to progressive rupture of one or more steel cords, while some others continue to slip within the matrix. The occurrence of complete debonding (i.e., debonding along the entire bonded length) for some steel cords is confirmed by the occurrence of nonzero values of the free end slip *s_F_* close to the attainment of σ* [28].

The representative response of CRM-masonry joints (Figure 3), which fail due to debonding of the composite grid with extensive cracking of the matrix [11,14], is characterized by an initial linear behavior followed by a non-linear branch associated with matrix cracking [11]. Indeed, after the initial linear branch, matrix cracks orthogonal to the direction of the applied load in the external matrix layer are induced by stress concentration at the transversal yarn (i.e., weft yarns, Table 1) locations and determine drops in the load response. These cracks are typical of inorganic-matrix reinforcements where the fiber reinforcement has longitudinal and transversal yarns firmly connected, which allows for a contribution of the transversal yarns to the applied load [49]. With increasing global slip, the cracks propagate from the external toward the internal matrix layer. Failure of the specimen generally occurs due to sudden detachment of the external matrix layer and/or of the entire reinforcement strip without damage of the masonry substrate.

For all inorganic-matrix reinforcements investigated in this study, debonding at the matrix–substrate interface may occur (Figure 3), with no (or minor) damage of the substrate. This debonding mode is caused by poor bond between matrix and substrate or by inadequate surface preparation.

In the following sections, the σ-*g* responses of the tested specimens are analyzed and discussed to shed light on the influence of wet–dry cycles on the specimen behavior and failure mode. 

### 3.1. Visual Inspection and Failure Modes 

**Figure 4:**
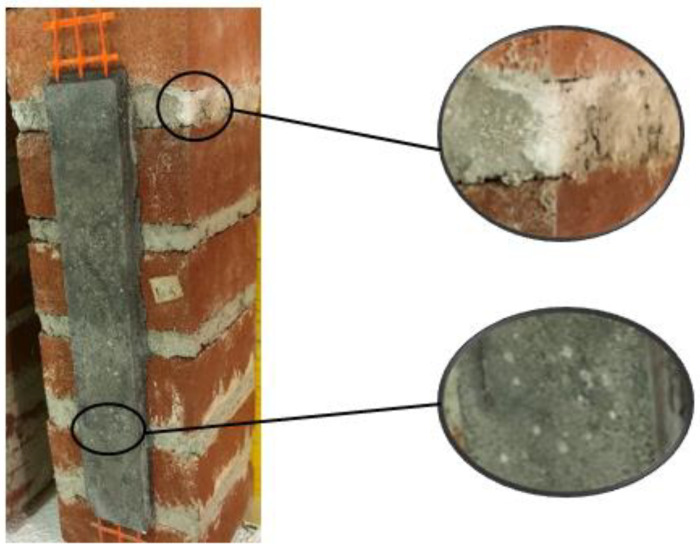
Salt efflorescence in specimen DS_300_50_G_W/D_5.

At the end of the conditioning period, the specimens were visually inspected. Small salt efflorescences were detected on the matrix, bricks, and mortar, as shown in Figure 4. Since the water used to condition the specimens was tap water and no salt was added to the solution, the efflorescences were caused by the salt present in small concentrations within the utilized materials. The presence of salt was also observed at the matrix–substrate interface after debonding. However, no sign of severe deterioration (e.g., flacking or crumbling) was observed on the specimens. Similar findings were also reported by Franzoni et al. [45]. 

Four different failure modes, illustrated in boxes (a) to (d) of Figure 3, were observed. They were named following the notation *J_z_*, where *J* indicates the failure mode (*D =* debonding, *R* = fiber rupture, and *M* = mixed failure mode) and subscript *Z* indicates the position of failure (*ms* = at the matrix–substrate interface and *mf* = at the matrix–fiber interface). Failure mode *D_ms_* [see box (a) in Figure 3] was characterized by debonding of the composite strip at the matrix–substrate interface, with no masonry damage. Failure mode *D_mf_* was characterized by slippage of the fiber within the matrix [see box (b) in Figure 3], while in failure mode *R* the textile ruptured within or outside the bonded length [see box (c) in Figure 3]. This failure mode was always preceded by matrix–fiber debonding, leading to a mixed failure mode *MD_mf_R*. Finally, mixed debonding failure at the matrix–fiber interface and matrix–substrate interface (*MD_mf_D_ms_*) was observed [see box (d) of Figure 3] for CRM reinforcement, which was followed by textile rupture for some specimens (*MD_mf_D_ms_R*). 

The failure modes observed are reported in Table 2 for each specimen and are discussed in the following sections. 

### 3.2. Carbon FRCM-Masonry Joints

**Figure 5:**
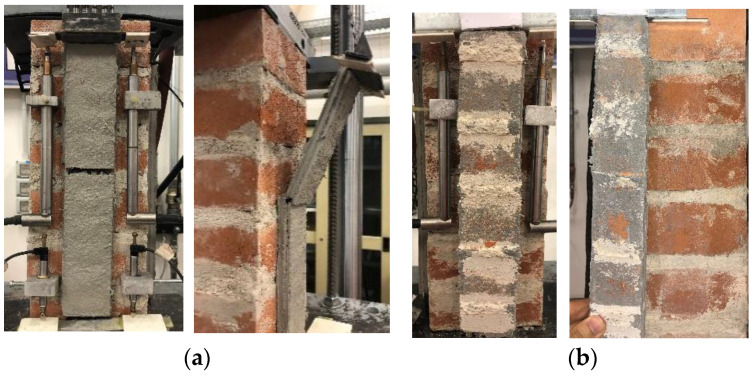
Failure modes of specimen (**a**) DS_300_50_C_1 (*MD_mf_D_ms_*) and (**b**) DS_300_50_C_W/D_1 (*D_ms_*).

Two failure modes were observed in the reference (non-strengthened) carbon FRCM-masonry joints. The most common failure mode was *D_mf_*, which was observed in 3 specimens (see Table 2). Specimen DS_300_50_C_1 showed a mixed failure mode *MD_mf_D_ms_*. First, matrix–fiber debonding occurred, which was followed by the opening of a matrix crack at approximately 140 mm from the loaded end (Figure 5a). This crack triggered the sudden detachment of the composite strip from the substrate at the loaded end, whereas slippage of the textile within the matrix for the composite portion still bonded to the masonry substrate continued. 

After conditioning, two failure modes were observed. Specimens DS_300_50_C_W/D_1 and 4 showed failure mode *D_ms_* (Figure 5b), while the remaining specimens showed matrix–fiber debonding followed by textile rupture (*MD_mf_R*).

**Figure 6:**
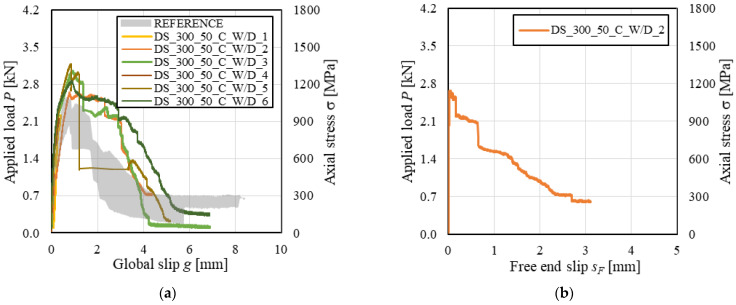
(**a**) *P-g* and (**b**) *P-s_F_* responses of FRCM-masonry joints with carbon fiber textile.

The applied load *P* (and axial stress σ)-global slip *g* responses of conditioned carbon FRCM-masonry joints are shown in Figure 6a, where the envelope of load responses of reference specimens is also reported for comparison. Conditioned specimens provided a load response consistent with that of corresponding reference specimens, except for specimens DS_300_50_C_W/D_1 and 4 that failed due to matrix–substrate debonding during the *P*-*g* ascending branch. For specimens DS_300_50_C_W/D_2, 3, 5, and 6, the *P*-*g* response showed sudden drops during the post-peak response, which can be attributed to failure of carbon fibers within the bonded length (no failure of bare fibers was observed). These load drops can be observed also in the applied load *P* (and axial stress σ)-free end slip *s_F_* response of specimen DS_300_50_C_W/D_2 (Figure 6b). Figure 6b showed that *s_F_* remained approximately null up to the attainment of P*, when it started increasing. This indicates that the entire matrix–fiber interface was engaged in the stress transfer mechanism when P* was reached. The constant applied stress at the end of the *P*-*s_F_* response confirms the presence of friction at the matrix–fiber interface and that debonding occurred along the entire bonded length. A constant applied stress branch was also observed at the end of the test of specimen DS_300_50_C_W/D_6. This friction stress was lower than that of specimen DS_300_50_C_W/D_2, which could be attributed to higher damage and failure of carbon fiber filaments in specimen DS_300_50_C_W/D_6 than in specimen DS_300_50_C_W/D_2.

**Figure 7:**
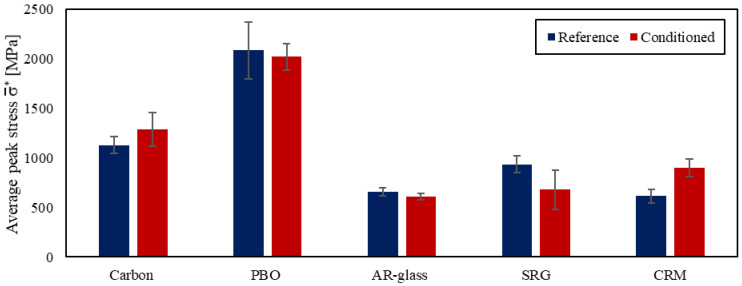
Comparison of average peak stresses of reference and conditioned specimens.

In Figure 7, average peak stresses ${\bar{\sigma }}^{*}$ of reference and conditioned specimens are compared (the standard deviation is depicted with error bars). In general, the wet–dry conditioning did not adversely affect the capacity of carbon FRCM-masonry joints. In fact, the peak stress of conditioned specimens was higher than the average peak stress of reference specimens (except for DS_300_50_C_W/D_1 and 4, see Table 2), which led to an increase of 14% of ${\bar{\sigma }}^{*}$ of conditioned specimens with respect to reference specimens. This increase of ${\bar{\sigma }}^{*}$ was attributed to the continuation of the matrix hydration process, which improved the matrix mechanical properties and matrix–fiber bond capacity. However, an increase of the coefficient of variation (CoV) from 8.7% to 14.7% was observed after conditioning, as a result of the different failure modes reported. 

### 3.3. PBO FRCM-Masonry Joints

**Figure 8:**
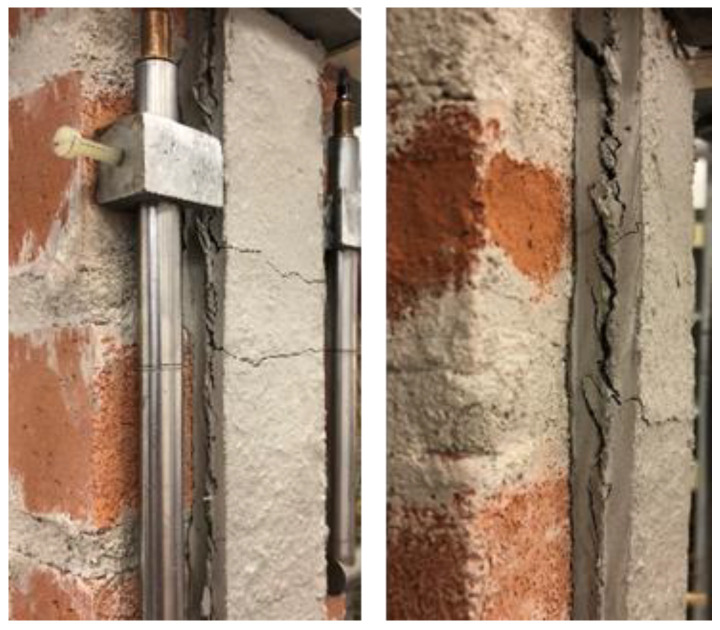
Opening of matrix transversal cracks and longitudinal cracks at the matrix internal–external layer interface in specimen DS_300_50_P_3 [28].

All reference PBO FRCM-masonry joints failed due to debonding at the matrix–fiber interface (*D_mf_*). Debonding of the textile from the embedding matrix was accompanied by the opening of transversal cracks in the matrix external layer, along with the opening of a longitudinal crack at the internal–external matrix layer interface (matrix delamination) (Figure 8). After the wet–dry cycles, all specimens showed failure due to debonding at the matrix–fiber interface followed by textile rupture (*MD_mf_R*). In specimens DS_300_50_P_W/D_1 and 5, textile rupture occurred outside the bonded length, while in specimens DS_300_50_P_W/D_2, 3, and 6, textile telescopic failure occurred within the bonded length [6]. The result of specimen DS_300_50_C_W/D_4 was disregarded due to machine issues during the tests. 

The results obtained confirmed the degradation of PBO fibers directly exposed to humid and warm environments reported in the literature [50]. However, in real applications, the PBO textile would be embedded in the matrix, which may prevent or limit the fiber degradation. Further studies are needed to clarify the long-term behavior of PBO FRCM using tests where the textile is fully embedded within the matrix.

**Figure 9:**
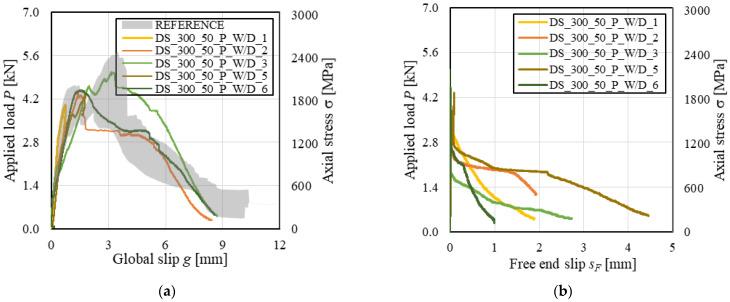
(**a**) *P-g* and (**b**) *P-s_F_* responses of FRCM-masonry joints with PBO fiber textile.

The *P-g* (and σ-*g*) responses of conditioned specimens are provided in Figure 9a along with the envelope of reference specimen responses. The wet–dry cycles did not affect the load response ascending branch, which was consistent for reference and conditioned specimens. However, the degradation of PBO fibers, which led to textile rupture in conditioned specimens, influenced the load response. As commonly observed in inorganic-matrix composites including bare (i.e., not impregnated) textiles, textile rupture occurred with progressive failure of single fiber filaments rather than with a sudden failure of all filaments at the same time [6]. For specimens DS_300_50_P_W/D_1 and 5, in which textile rupture occurred outside the bonded length, the global slip remained constant as the applied load decreased after P* due to progressive fiber filaments rupture (Figure 9a). With decreasing the applied load, the textile within the bonded length recovered the elastic deformation and tended to close the transversal matrix crack. The same phenomenon was observed for transversal matrix cracks in specimens DS_300_50_P_W/D_2, 3, and 6 that, after the occurrence of textile telescopic failure within the bonded length, tended to close. In these specimens, *g* increased with decreasing *P* after P* as the fiber filaments still not ruptured were pulled out of the matrix.

Figure 9b shows the applied load *P*-free end slip *s_F_* response of conditioned PBO FRCM-masonry joints. For all specimens, non-null values of *s_F_* were observed during the load response descending branch after a significant decrease of *P*. This confirms the presence of the telescopic failure, which determined rupture of some fiber filaments within the bonded length while others remained undamaged and slipped with respect to the matrix.

Figure 7 illustrates the effect of the wet–dry cycles on the average peak stress. Although a 7% decrease of ${\bar{\sigma }}^{*}$ can be observed, this variation is not significant since it falls within the data scatter (CoV = 13.7% and 7.8% for reference and conditioned specimens, respectively). Nonetheless, the PBO fibers were damaged by direct exposure to the humid and warm environment, which affected the failure mode of FRCM-masonry joints. Therefore, further studies are needed to clarify the long-term behavior of the PBO FRCM composite when the fibers are fully embedded within the matrix, possibly investigating the role of matrix cracks that could expose the fiber to the aggressive environment [51].

### 3.4. AR Glass FRCM-Masonry Joints

**Figure 10:**
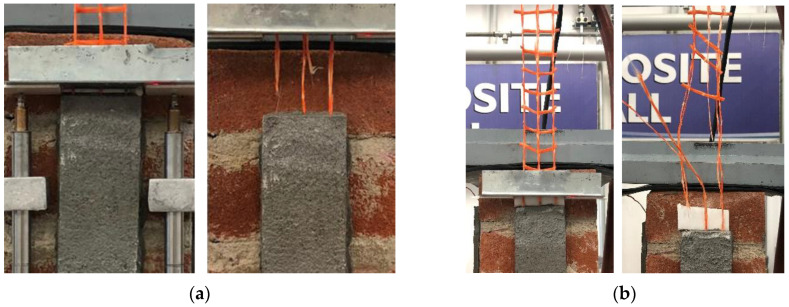
*MD_mf_R* failure (**a**) within the bonded length (specimen DS_300_50_G_W/D_1) and (**b**) outside the bonded length (specimen DS_300_50_G_W/D_2).

All reference and conditioned specimens failed due to debonding at the matrix–fiber interface followed by textile rupture (*MD_mf_R*). Textile rupture occurred within the bonded length for most of the specimens (Figure 10a), though for specimen DS_300_50_G_W/D_2, 4, and 6, progressive failure of single bundles outside the bonded length was observed (Figure 10b).

**Figure 11:**
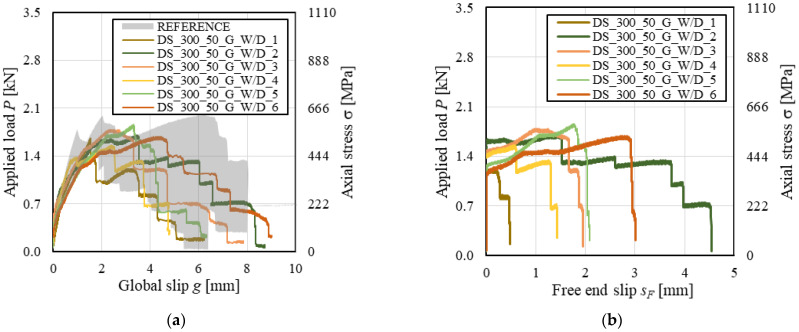
(**a**) *P-g* and (**b**) *P-s_F_* responses of FRCM-masonry joints with glass fiber textile.

The load responses of reference (envelope) and conditioned specimens are provided in Figure 11a. Both reference and conditioned specimens showed a similar ascending branch, which was characterized by an initial linear behavior followed by a non-linear branch. Close to the peak load, one or more fiber bundles failed, causing a sudden load drop. Failure of single bundles was attributed to the uneven distribution of the applied load across the composite strip width, which in turn is affected by the randomly distributed matrix–fiber interface properties. The presence of the textile coating (see Section 2.1) allowed for the contemporary failure of all fiber filaments in the same bundle (Figure 10). As the applied load decreased after the attainment of P*, numerous load drops associated with progressive failure of textile bundles were observed in the load response. 

Figure 11b shows the *P*-*s_F_* responses for the specimens tested. The presence of non-null values of *s_F_*, which started increasing before the attainment of peak load, indicates that the entire bonded length was engaged in the stress transfer mechanism. The presence of friction at the matrix-fiber interface could not be confirmed due to the eventual rupture of textile and consequent absence of a residual applied stress at the end of the test. For specimens DS_300_50_G_W/D_1, rupture of one of the edge bundles induced a significant rotation of the L-shaped plate attached to the textile at the free end, which in turn determined values of *s_F_* lower than those of other specimens.

The effect of the wet–dry cycles on ${\bar{\sigma }}^{*}$ is presented in Figure 7, where a 7% decrease from reference to conditioned specimens can be observed. Compared with other FRCM studied, the results of the AR glass FRCM-masonry joints presented the lowest scatter (CoV = 6.4% and 5.6% for reference and conditioned specimens, respectively). The low dispersion of results can be attributed to the failure mode observed, which was the same for all specimens and was controlled by the failure of the textile, i.e., it was not affected by the random distribution of the matrix–fiber bond properties. 

### 3.5. SRG-Masonry Joints 

**Figure 12:**
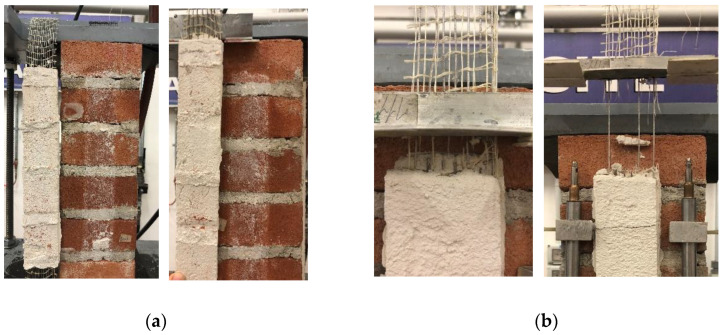
Failure modes of SRG-masonry joints: (**a**) *D_ms_* (specimen DS_300_50_S_W/D_3) and (**b**) *MD_mf_R* (specimen DS_300_50_S_W/D_6).

In the reference specimens, debonding of the composite strip from the substrate (*D_ms_*, 2 specimens) and matrix–fiber debonding followed by textile rupture (*MD_mf_R*, 2 specimens) were observed (see Table 2). After conditioning, all specimens showed failure mode *D_ms_* (Figure 12a) except specimen DS_300_50_S_W/D_6, which failed with mode *MD_mf_R* (Figure 12b). This may indicate a decrease of matrix–substrate bond properties in conditioned specimens.

**Figure 13:**
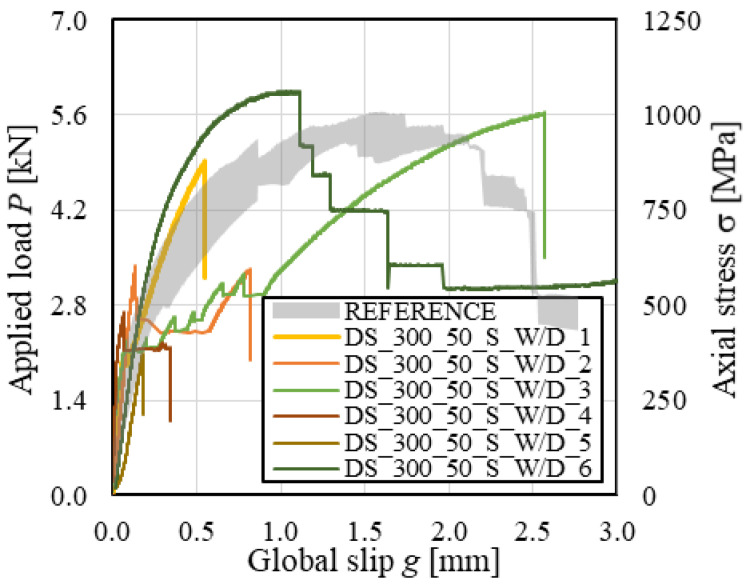
*P-g* responses of SRG-masonry joints.

The *P-g* (and σ-*g*) responses of conditioned SRG-masonry joints are presented in Figure 13, where the envelope of reference specimen load responses is also provided for comparison. All specimens showed an initial linear branch, which was suddenly interrupted due to detachment of the composite strip in specimens DS_300_50_S_W/D_2, 4, and 5. In specimens DS_300_50_S_W/D_1 and 3, after the initial linear branch, the load response became non-linear due to the occurrence of micro-cracking at the matrix–fiber interface until sudden *D_ms_* failure occurred. Only in specimen DS_300_50_S_W/D_6, the matrix–substrate bond was sufficient to attain tensile rupture of some steel cords, as shown by subsequent load drops in the load response (Figure 13). In this specimen, a residual applied load was attained at completion of the test due to the friction between the remaining unruptured steel cords and the embedding matrix. It should be noted that in some cases (e.g., specimens DS_300_50_S_W/D_1 and 3) cracks occurred in the matrix external layer and eventually propagated toward the masonry substrate, which may have contributed in triggering the matrix–substrate debonding.

Figure 7 illustrates the effect of wet–dry cycles on the average peak stress obtained. A decrease of 27% of ${\bar{\sigma }}^{*}$ can be noticed, while the CoV increased from 9.5% to 30.4% from reference to conditioned specimens, respectively. Comparing specimens that reported the same failure mode, ${\bar{\sigma }}^{*}$ of conditioned specimens was 29% lower than that of reference specimens with failure mode *D_ms_*, while no significant variation of peak load was observed for specimens with failure mode *MD_mf_R*. These results suggest that wet–dry cycles affected the matrix–substrate interface rather than the composite itself. However, due to the significant scatter observed, further results are needed to confirm this observation. 

### 3.6. CRM-Masonry Joints 

**Figure 14:**
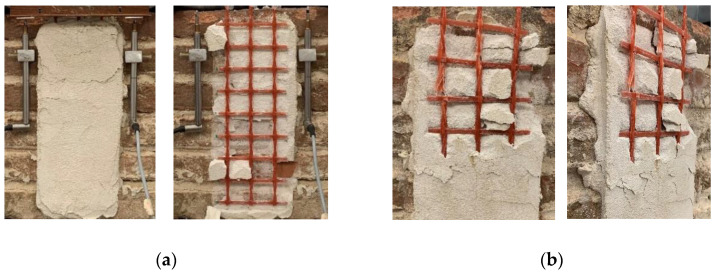
CRM-masonry joint failure modes: (**a**) *MD_mf_D_ms_* (specimen DS_290_120_CRM_W/D_2) and (**b**) *MD_mf_D_ms_R* (specimen DS_290_120_CRM_W/D_1).

All reference CRM-masonry joints presented a similar failure mode, which was characterized by matrix–fiber and matrix–substrate debonding accompanied by extensive cracking of the matrix external layer (*MD_mf_D_ms_*). This failure mode is typical of open-mesh reinforcements with longitudinal and transversal yarns firmly connected [49] (see Section 3). Conditioned specimens showed the same failure mode, which for specimens DS_290_120_CRM_W/D_1, 3, 5, and 6 was also associated with the eventual rupture of grid longitudinal yarns (*MD_mf_D_ms_R*). Figure 14a shows failure of specimen DS_290_120_CRM_W/D_2, while Figure 14b that of specimen DS_290_120_CRM_W/D_1. As shown in Figure 14b, grid failure occurred close to the longitudinal–transversal yarn joints, due to the presence of stress concentrations [49].

**Figure 15:**
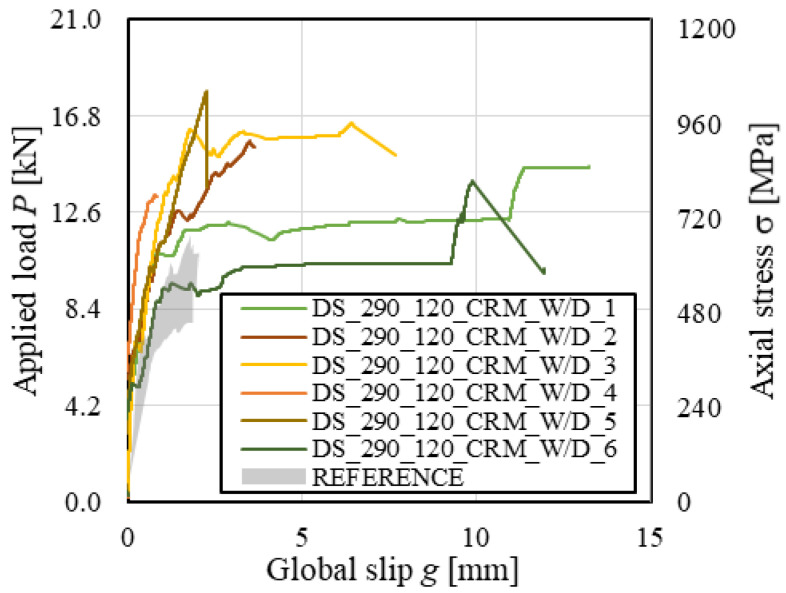
*P-g* responses of CRM-masonry joints.

The *P-g* (and σ-*g*) responses of conditioned specimens are presented in Figure 15, where the reference load responses are provided as an envelope. Both reference and conditioned specimens showed an initial linear branch followed by a non-linear behavior associated with micro-cracking at the matrix–fiber interface. When transversal cracks occurred in the matrix due to the stress concentration induced by the transversal yarns, load drops were noticed in the *P-g* response. These drops were associated with sudden increases of global slip *g* caused by the release of elastic energy stored in the composite grid. While reference specimens reported failure mode *MD_mf_D_ms_*, conditioned specimens DS_290_120_CRM_W/D_1, 3, and 6 attained grid rupture. Due to the uneven distribution of the applied load among the longitudinal yarns, subsequent rupture of individual grid yarns always occurred in these specimens. This determined sudden increases of *g*, which was significantly affected by the rotation of the L-shaped aluminum plate attached to the grid caused by yarn failure. However, in specimen DS_290_120_CRM_W/D_5, all longitudinal yarns failed at the same time, which allowed for attaining the highest peak load. It should be noted that grid tensile rupture was not caused by degradation of the textile. In fact, stresses associated with grid rupture were consistent with if not higher than the bare grid tensile strength (see Table 1).

Conditioned specimens generally showed higher peak loads than reference specimens. The average peak stress increased by 47% from reference to conditioned specimens (Figure 7), with a similar CoV (CoV = 11.7% and 9.7% for reference and conditioned specimens, respectively). This effect was attributed to curing of the lime-based mortar induced by wet–dry cycles. The mechanical properties of the conditioned matrix increased the slope of the initial linear branch of *P*-*g* curves and provided a better matrix-grid interlocking, thus increasing the peak load obtained.

## 4. Conclusions 

In this paper, the long-term bond behavior of various inorganic-matrix reinforcements was studied. Carbon, PBO, and AR glass FRCM-, SRG-, and CRM-masonry joints were exposed to 50 wet–dry cycles and then tested using a single-lap direct shear test set-up. Results allowed for drawing the following conclusions:

- Wet–dry cycles promoted the occurrence of salt efflorescence on the external surfaces of matrix, bricks, and at the matrix–substrate interface, which was attributed to the presence of salt within the utilized materials.
- In carbon FRCM-masonry joints, the failure mode was in some cases shifted from debonding at the matrix–fiber interface in reference specimens to debonding at the matrix–substrate interface followed by textile rupture for conditioned specimens. However, the average peak stress increased by 14% after conditioning, which was attributed to the continuation of the hydration process.
- In conditioned PBO FRCM-masonry joints, matrix–fiber debonding was accompanied by failure of PBO textile outside or within the bonded length, which was caused by damage of the textile due to direct exposure to humid and warm conditions. However, no significant variation of the average peak stress was observed.
- Reference and conditioned AR glass FRCM-masonry joints showed a consistent behavior and the same failure mode due to matrix–fiber debonding and eventual textile rupture, with similar average peak stresses.
- Wet-dry cycles affected the behavior of SRG-masonry joints. In almost all conditioned specimens, failure due to detachment of the composite strip from the substrate was observed and the average peak stress for these specimens decreased by 29%.
- In CRM-masonry joints, wet–dry cycles promoted curing of the lime-based matrix, which in turn was responsible for a 47% increase of average peak stress with respect to that of reference specimens. Furthermore, grid tensile failure was attained in conditioned specimens at stresses consistent with the bare grid tensile strength.

No research has been done so far to study the combined effect of aggressive environment exposure and sustained load on the performance of the inorganic-matrix reinforcement and reinforcement–substrate bond. Furthermore, still limited information on the effect of freeze–thaw cycles, alkaline environments, sulfate attack, hygrothermal conditions, and other exposures is available in the literature. A huge effort should be made in this direction to promote the safe and reliable applications of these promising reinforcing systems.

## Figures and Tables

**Table 2:** Results of direct shear single-lap tests.

Specimen Name	*L* [mm]	*b*_1_ [mm]	*n* [-]	*A* [mm^2^]	P* [kN]	${\bar{P}}^{*}(\mathrm{CoV})$ [kN] (%)	σ* [MPa]	${\bar{\sigma }}^{*}(\mathrm{CoV})$ [MPa] (%)	FM
DS_300_50_C_1 ^¤^	300	50	5	2.35	2.42	2.37 (8.70)	1152	1130 (8.70)	*MD_mf_D_ms_*
DS_300_50_C_2 ^¤^					2.46		1171		*D_mf_*
DS_300_50_C_3 ^¤^					2.58		1229		*D_mf_*
DS_300_50_C_4 ^¤^					2.03		967		*D_mf_*
DS_300_50_C_W/D_1					2.23	2.71 (14.68)	1062	1290 (14.68)	*D_ms_*
DS_300_50_C_W/D_2 ^§^					2.67		1269		*MD_mf_R*
DS_300_50_C_W/D_3					3.08		1468		*MD_mf_R*
DS_300_50_C_W/D_4					2.17		1034		*D_ms_*
DS_300_50_C_W/D_5					3.21		1531		*MD_mf_R*
DS_300_50_C_W/D_6					2.89		1376		*MD_mf_R*
DS_300_50_P_1 ^¤^	300	50	5	2.30	5.67	4.80 (13.68)	2465	2086 (13.68)	*D_mf_*
DS_300_50_P_2 ^¤^					4.66		2026		*D_mf_*
DS_300_50_P_3 ^¤^					5.01		2178		*D_mf_*
DS_300_50_P_4 ^¤^					3.85		1674		*D_mf_*
DS_300_50_P_W/D_1 ^§^					4.01	4.46 (7.80)	1743	1939 (7.80)	*MD_mf_R*
DS_300_50_P_W/D_2 ^§^					4.37		1900		*MD_mf_R*
DS_300_50_P_W/D_3 ^§^					5.07		2205		*MD_mf_R*
DS_300_50_P_W/D_5 ^§^					4.35		1891		*MD_mf_R*
DS_300_50_P_W/D_6 ^§^					4.49		1954		*MD_mf_R*
DS_300_50_G_1 ^¤^	300	50	3	3.15	1.77	1.85 (6.43)	560	585 (6.43)	*MD_mf_R*
DS_300_50_G_2 ^¤^					1.69		535		*MD_mf_R*
DS_300_50_G_3 ^¤^					1.95		619		*MD_mf_R*
DS_300_50_G_4 ^¤^					1.97		626		*MD_mf_R*
DS_300_50_G_W/D_1 ^§^					1.66	1.71 (5.58)	528	542 (5.58)	*MD_mf_R*
DS_300_50_G_W/D_2 ^§^					1.70		540		*MD_mf_R*
DS_300_50_G_W/D_3 ^§^					1.79		568		*MD_mf_R*
DS_300_50_G_W/D_4 ^§^					1.56		494		*MD_mf_R*
DS_300_50_G_W/D_5 ^§^					1.86		590		*MD_mf_R*
DS_300_50_G_W/D_6 ^§^					1.69		535		*MD_mf_R*
DS_300_50_S_1 ^¤^	300	50	7	5.60	4.36	5.14 (9.47)	793	934 (9.47)	*D_ms_*
DS_300_50_S_2 ^¤^					5.28		961		*D_ms_*
DS_300_50_S_3 ^¤^					5.2		946		*MD_mf_R*
DS_300_50_S_4 ^¤^					5.7		1037		*MD_mf_R*
DS_300_50_S_W/D_1					4.91	3.73 (30.38)	894	679 (30.38)	*D_ms_*
DS_300_50_S_W/D_2					3.38		614		*D_ms_*
DS_300_50_S_W/D_3					4.02		732		*D_ms_*
DS_300_50_S_W/D_4					2.71		493		*D_ms_*
DS_300_50_S_W/D_5					2.09		380		*D_ms_*
DS_300_50_S_W/D_6					5.26		957		*MD_mf_R*
DS_G_290_120_Y_1 ^‡^	290	120	3	17.13	10.71	10.52 (11.72)	626	615 (11.72)	*MD_mf_D_ms_*
DS_G_290_120_Y_2 ^‡^					11.74		687		*MD_mf_D_ms_*
DS_G_290_120_Y_3 ^‡^					8.30		485		*MD_mf_D_ms_*
DS_G_290_120_Y_4 ^‡^					11.06		647		*MD_mf_D_ms_*
DS_G_290_120_Y_5 ^‡^					10.03		587		*MD_mf_D_ms_*
DS_G_290_120_Y_6 ^‡^					11.30		661		*MD_mf_D_ms_*
DS_290_120_CRM_W/D_1					15.32	15.44 (9.73)	894	901 (9.73)	*MD_mf_D_ms_R*
DS_290_120_CRM_W/D_2					15.69		916		*MD_mf_D_ms_*
DS_290_120_CRM_W/D_3					16.47		961		*MD_mf_D_ms_R*
DS_290_120_CRM_W/D_4					13.34		779		*MD_mf_D_ms_*
DS_290_120_CRM_W/D_5					17.85		1042		*MD_mf_D_ms_R*
DS_290_120_CRM_W/D_6					13.95		814		*MD_mf_D_ms_R*

^¤^ data from [28]; **^‡^** data from [11]; **^§^** *s_F_* was measured.

## Data Availability

The data presented in this study are available on request from the corresponding author. The data are not publicly available because the research is still ongoing.
